# Acupuncture Stimulation Attenuates Impaired Emotional-Like Behaviors and Activation of the Noradrenergic System during Protracted Abstinence following Chronic Morphine Exposure in Rats

**DOI:** 10.1155/2014/216503

**Published:** 2014-01-09

**Authors:** Bombi Lee, Bong-Jun Sur, Insop Shim, Dae-Hyun Hahm, Hyejung Lee

**Affiliations:** ^1^Acupuncture and Meridian Science Research Center, College of Oriental Medicine, Kyung Hee University, 1 Hoegi-dong, Dongdaemun-gu, Seoul 130-701, Republic of Korea; ^2^The Graduate School of Basic Science of Oriental Medicine, College of Oriental Medicine, Kyung Hee University, Seoul 130-701, Republic of Korea

## Abstract

The purpose of this study was to evaluate whether acupuncture stimulation attenuates withdrawal-induced behaviors in the rats during protracted abstinence following chronic morphine exposure. To do this, male rats were first exposed to morphine gradually from 20 to 100 mg/kg for 5 days, and subsequently naloxone was injected once to extend despair-related withdrawal behaviors for 4 weeks. Acupuncture stimulation was performed once at the SP6 (Sanyinjiao) acupoint on rat's; hind leg for 5 min during protracted abstinence from morphine. The acupuncture stimulation significantly decreased despair-like behavior deficits in the forced swimming test and low sociability in the open-field test as well as increased open-arm exploration in the elevated plus maze test in the last week of 4-week withdrawal period. Also the acupuncture stimulation significantly suppressed the increase in the hypothalamic corticotropin-releasing factor (CRF) expression, the decrease in the tyrosine hydroxylase expression in the locus coeruleus, and the decrease in the hippocampal brain-derived neurotrophic factor mRNA expression, induced by repeated injection of morphine. Taken together, these findings demonstrate that the acupuncture stimulation of SP6 significantly reduces withdrawal-induced behaviors, induced by repeated administration of morphine in rats, possibly through the modulation of hypothalamic CRF and the central noradrenergic system.

## 1. Introduction

Morphine, a strong pain reliever, is widely used to treat moderate to severe pain as well as a number of other pathological indications. The continuous use of morphine causes drug craving, tolerance to opiate analgesia, and a withdrawal syndrome, particularly a relapse into drug-seeking behavior, when the drug is discontinued [1]. Accordingly, the abuse of morphine and subsequent withdrawal from it result in an aversive emotional state and debilitating physical symptoms including restlessness, high emotionality, and lowered mood [2]. Many studies have demonstrated that protracted morphine abstinence following spontaneous withdrawal causes emotion-related disorders in humans and corresponding behavioral responses in animals [2, 3]. Importantly, the depression and anxiety that occur during morphine addition and morphine abstinence often lead to a relapse in humans [4]. The depression and anxiety associated with morphine withdrawal can be alleviated by the administration of antidepressant or anxiolytic drugs such as fluoxetine or agmatine [5, 6]. However, most antidepressants are not very effective against the wide variety of complex depressive symptoms, and most are associated with serious side effects such as drowsiness, dryness of the mouth, headache, nausea, and sexual dysfunction [7, 8]. Thus, it is crucial to identify alternative treatments that balance antidepressant therapeutic effects and with minimal side effects [9]. Recently, acupuncture, a traditional therapy that has become increasingly popular in Western countries, has been utilized to treat psychiatric disorders including withdrawal symptoms associated with morphine use [10]. A number of studies have found that acupuncture is an effective remedy for depression and anxiety and may be as effective as antidepressant drugs [11].

In East Asian nations, acupuncture has been widely used as a therapeutic method to treat many psychosomatic disorders including major depression, stress, and drug abuse [12]. Its therapeutic effects and mechanisms have been investigated in both clinical and animal studies. This alternative therapy is known to modulate biochemical balances in the central nervous system (CNS) and to maintain homeostasis [13]. SP6 (Sanyinjiao) is one of the most commonly used acupoints in acupuncture treatment to alleviate psychic and psychosomatic dysfunction such as depressive symptoms and anxiety disorders [14]. Recent animal studies have found that acupuncture and low frequency electroacupuncture stimulation exhibit antidepressant-like activity in the forced swimming test (FST), reduce anxiety-like behavior in the elevated plus maze (EPM) test, and restore the expression levels of neuropeptide Y and c-Fos in the brain [15–17]. However, the underlying mechanism of acupuncture in the treatment of psychiatric disorders, including the withdrawal-induced despair-related, anxiety-like, and aberrant social behaviors associated with morphine use, has not been examined in animal models.

The aim of the present study was to investigate the effects of acupuncture stimulation on despair-related, anxiety-like, and social behaviors in rats exposed to repeated morphine administration. Additionally, withdrawal-induced behaviors associated with morphine discontinuation were evaluated using the FST, the EPM test, and the open field test (OFT) following a short (1 week) or prolonged (4 weeks) period of abstinence subsequent to chronic morphine exposure. This investigation also aimed to identify the underlying mechanisms of this process to elucidate how withdrawal-like behaviors associated with protracted abstinence from chronic morphine were associated with the noradrenergic system in the brain.

## 2. Methods

### 2.1. Animals

Adult male Sprague-Dawley (SD) rats weighing 260–280 g (6 weeks-old) were obtained from Samtako Animal Co. (Seoul, Rebuplic of Korea). The rats were housed in a limited access rodent facility with up to five rats per polycarbonate cage. The room controls were set to maintain the temperature at 22 ± 2°C and the relative humidity at 55 ± 15%. Cages were lit by artificial light for 12 h each day. Sterilized drinking water and standard chow diet were supplied ad libitum to each cage during the experiments. The animal experiments were conducted in accordance with the National Institutes of Health Guide for the Care and Use of Laboratory Animals (NIH Publications no. 80-23), revised in 1996, and were approved by the Kyung Hee University Institutional Animal Care and Use Committee. All animal experiments began at least 7 days after the animals arrived.

### 2.2. Experiment and Animal Groups

#### 2.2.1. General Procedure: Experiment 1

*Induction of Morphine Dependence*. Morphine dependence was induced by the repeated administration of morphine twice a day (09:30 and 19:30 h) during 5 consecutive days. Animals received two daily applications of increasing doses of morphine according to the following treatment schedule: day 1, 2 × 20 mg/kg; day 2, 2 × 40 mg/kg; day 3, 2 × 60 mg/kg; day 4, 2 × 80 mg/kg; day 5, 2 × 100 mg/kg, as described previously [3]. The final morphine injection (single 100 mg/kg) was given in the morning of the sixth day. As a vehicle control, animals were subcutaneously given (s.c.) in equivalent volumes with saline solution. To confirm the induction of physical dependence, on the sixth day of treatment, 2 h after the final morphine injection, rats were administered opioid antagonist naloxone (0.1 mg/kg, i.p.) in order to precipitate withdrawal in morphine dependence (*n* = 4/group). The antagonist dosage was selected by the study of Castilho et al. [18]. Morphine hydrochloride and naloxone hydrogen chloride (Sigma-Aldrich Chemical Co., St. Louis, MO, USA) were purchased from the standard commercial suppliers. Morphine hydrochloride and naloxone hydrogen chloride were dissolved in 0.9% physiological saline and were freshly prepared right before every experiment. Rats were evaluated to one of the well-known physical withdrawal signs test. Physical withdrawal signs including jumps, paw tremors, shakes, and sniffing were scored for 25 min, according to procedures described previously by Goeldner et al. [3].

**Figure 1:**
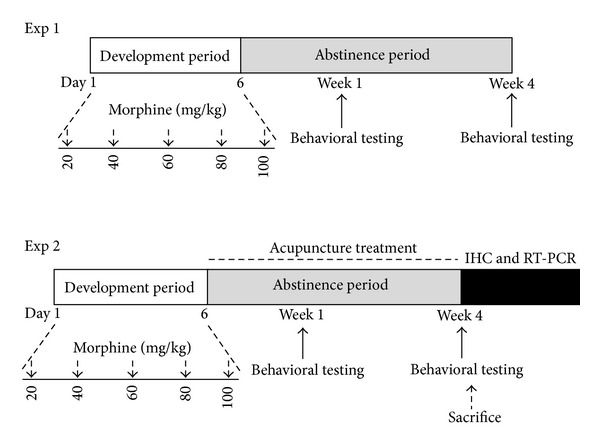
Experimental schedule for study of morphine withdrawal-induced depression- and anxiety-like behaviors in rats.

*Development of Abstinence.* Rats were maintained drug free after chronic treatment and experienced withdrawal in their home cages. One or 4 weeks following the final morphine injection, despair-related, anxiety-like, and aberrant social behaviors in rats exposed to abstinence were evaluated using the FST, the EPM test, and the OFT. The entire experimental schedule of all drug administration and behavioral examinations are shown in Figure 1. The following parameters were measured to monitor the effects of the development of psychosomatic disorders by repeated morphine administration: changes of body weight gains and sucrose intake. After the behavioral testing and body weighting, rats were sacrificed and brain tissues were immediately collected for experiments or stored at −70°C for later use.

#### 2.2.2. Experiment 2

This study was designed to explore the efficacy of acupuncture stimulation for healing repeated morphine administration and withdrawal-induced despair-related, anxiety-like, and aberrant social behaviors in an animal model using behavioral and neurobiological methodologies. The withdrawal group following repeated morphine administration was given morphine (20, 40, 60, 80, 100 mg/kg-body weight, *s.c.*, MOR group, *n* = 7) twice a day for 5 consecutive days. One or 4 weeks following the final morphine injection, behavioral responses were tested during this period. The vehicle-treated rats (as a negative control in the development of addiction withdrawal model) were administered with saline (0.9% NaCl, *s.c.*) instead of morphine in the same way (SAL group, *n* = 7). The acupuncture-treated groups were divided as follows: Sanyinjiao (SP6) acupoint-stimulated plus morphine-treated group (MOR-SP, *n* = 7), Waiguan (TE5) acupoint-stimulated plus morphine-treated group (MOR-TE, *n* = 7), and nonacupoint (on the tail)-stimulated plus morphine-treated group (MOR-TA, *n* = 7).

### 2.3. Acupuncture Stimulation

Acupuncture stimulation was bilaterally performed every day for 5 min during protracted abstinence for 28 consecutive days, as the withdrawal phase. The acupuncture stimulation was performed as previously described [19]. In traditional oriental medicine, one of the most commonly used acupuncture points used for gynecological, fertility, digestive, urinary, sexual, and emotional disorders is SP6, or spleen 6 [20, 21]. Its name translates into English as three Yin intersections, reflective of it being a meeting point of the three Yin channels of the leg (spleen, liver, and kidney) [20, 21]. The SP6 point is located on the inside of the ankle, three fingerbreadths above the ankle bone [21, 22]. The acupoint on tail and the TE5 acupoint were selected as a nonacupoint and a comparison acupoint, respectively. As a comparable control acupoint, we also performed the stimulation to another acupoint, the TE5 acupoint, on a different (large intestine) meridian, and the triple-energizer channel, which is known to treat immune depression and pain/neuropathy of the arm [23]. To stimulate acupuncture in the fore or hind legs of rats, an acupuncture needle was inserted and manipulated by hand in the present study. Rats were seated on our thigh, and acupuncture stimulation was actually performed for 5 min. Rats were awake and gently calmed on our thigh with no physical restraint during acupuncture stimulation. During the acupuncture procedure, rats were gently handled entirely to minimize stress in the rats. We did not carry out sham acupuncture for control. The SAL group and the MOR group were handled for 5 min to a calming effect, instead of acupuncture stimulation, similar to what we described previously [19]. Therefore, the rats in control groups were handled under the same conditions but without acupuncture stimulation for 5 min and then returned to their cage. The sterilized disposable stainless steel acupuncture needles (0.30 × 2 mm, Suzhou Kangnian Medical Devices Co., Ltd., Shzhou, China) were inserted perpendicularly as deep as 2-3 mm at SP6 or TE5 acupoint. The depth of needle insertion at each acupoint was arbitrarily determined based on several previous studies [19].

### 2.4. Behavioral Testing

#### 2.4.1. Measurement of Locomotor Activity

During development of morphine dependence induced by the repeated injection of morphine during 5 consecutive days, the rats were individually housed in a rectangular container that was made of black polyethylene (30 × 30 × 45 cm) to provide the best contrast to the white rats in a dimly lit room equipped with a video camera above the center of the floor, and their locomotor activities (animal's movements) were then measured. The locomotor activity indicated by the speed and the distance of movements was monitored by a computerized video-tracking system using the S-MART program (Panlab Co., Barcelona, Spain). The animals were allowed to adapt to the container for 1 h before testing to acclimatize to the new environment. After 1 h of adaptation, the distance they traveled in the container was recorded for 1 h baseline period and for 1 hour after treatment. The locomotor activity was expressed in centimeters. The floor surface of each chamber was thoroughly cleaned with 70% ethanol between tests.

#### 2.4.2. Forced Swimming Test (FST)

FST, a representative behavioral test for depression, is frequently used to evaluate the activities of potential antidepressant drugs in rodent models. Forced immersion of rats in water for an extended period produces a characteristic behavior of immobility. The antidepressant treatments decrease the immobility behavior accompanied with an increase in the escape responses such as climbing and swimming. A transparent Plexiglas cylinder (20 cm diameter × 50 cm height) was filled up to a depth of 30 cm with water at 25°C. At this depth, rats could not touch the bottom of the cylinder with their tails or hind limbs. On day 1, the rats in all groups were trained for 15 min by placing them in the water-filled cylinder. On day 2, animals were subjected to 5 min of forced swim, and escape behaviors (climbing and swimming) were determined. The duration of immobility was scored during the 5 min test period. The animals' behavior was continuously recorded by experimenter-manual scoring during the testing session with an overhead video camera to tape behavior for later manual scoring. All of the behavioral scoring was done by a single trained rater, blind to experimental conditions. Several test sessions, chosen at random, were scored a second time by this rater to determine test-retest reliability, as previously described [24]. The scorer would rate the rat's behavior as one of the following three behaviors. Immobility behavior was calculated as the length of time in which the animal did not show escape responses (e.g., total time of the test minus time spent in climbing and swimming behaviors). The rats were judged to be immobile when they remained in the water without struggling and were making only those movements necessary to keep their head above water. Climbing behavior was defined as upward-directed movements of the forepaws along the side of the swim chamber, and swimming behavior was considered as movements throughout the swim chamber including crossing into another quadrant.

#### 2.4.3. Elevated Plus Maze (EPM) Test

The EPM test is a widely used behavioral test to assess anxiogenic or anxiolytic effects of pharmacological agents. Animals conduct anxiety-like behaviors usually show the reductions both in the number of entries and in the time spent in the open arms, along with an increase in the amount of time spent in the closed arms in the EPM. The elevated plus test was conducted. This apparatus consisted of two open arms (50 × 10 cm each), two closed arms (50 × 10 × 40 cm each), and a central platform (10 × 10 cm), arranged in a way such that the two arms of each type were opposite to each other. The maze was made from black Plexiglas and elevated 50 cm above the floor. Exploration of the open arms was encouraged by testing under indirect dim light (2 × 60 W). At the beginning of each trial, animals were placed at the centre of the maze, facing a closed arm. During a 5 min test period, the following parameters were recorded: (1) number of open arm entries, (2) number of closed arm entries, (3) time spent in open arms, and (4) time spent in closed arms. Entry by an animal into an arm was defined as the condition in which the animal has placed its four paws in that arm. The behavior in the maze was recorded using a video camera mounted on the ceiling above the center of the maze and relayed to the S-MART program (Panlab, Barcelona, Spain). Anxiety reduction, indicated by open arm exploration in the EPM, was defined as an increase in the numbers of entries into the open arms relative to total entries into either open or closed arm and an increase in the proportion of time spent in the open arms relative to total spending time in either open or closed arm. Total arm entries were also used as indicators of changes in locomotor activities of the rats.

#### 2.4.4. Open Field Test

For open field testing, the rats were individually housed to obtain reproducible results. Measurements of locomotor activities were conducted in a rectangular container (60 × 60 × 30 cm) equipped with a video camera above the center of the room, as described previously [19]. The walls and floor of the container were made of clear acrylic plastic and painted black. Locomotor activity was monitored by a video tracking system with S-MART program (Panlab Co., Barcelona, Spain). For doing this, each rat was placed to the centers of locomotor activity boxes, and the distance they traveled was recorded for 5 min. The number of line crossing (with all four paws) between the squares area was recorded for 5 min.

#### 2.4.5. Social Interaction

Social interaction test has been pharmacologically validated as an experimental paradigm to measure depression or anxiety [25]. This test was performed in a familiar situation (apparatus used was the same as for the OFT) under bright light. A pair of rats of same treatment condition and weight was placed simultaneously in the apparatus. Rats were placed in opposing corner of the open field apparatus and rats were allowed to explore the arena for 10 min. Active behavior was recorded when animals were running towards, grooming, sniffing, mounting, and crawling under the other rats. The total duration of active behavior and number of contacts were measured for each pair of animals. The analysis was performed by a person blind to the treatment.

### 2.5. Sucrose Intake

The sucrose intake test was performed as described previously with minor modifications [26]. For this test, rats were trained to consume a 1% sucrose solution prior to the start of the experiment. Briefly, 48 hours before the test, the rats were trained to adapt to 1% sucrose solution (w/v): two bottles of 1% sucrose solution were placed in each cage, and 24 hours later 1% sucrose solution in one bottle was replaced with water for 24 hours. After the adaption, rats were deprived of water and food for 10 hours. Sucrose preference test was conducted at 9:00 a.m. in which rats were housed in individual cages and were free to access to two bottles containing 100 mL of sucrose solution (1%, w/v) and 100 mL of water, respectively. After 3 hours, the volumes of consumed sucrose solution and water were measured. Sucrose preference was measured by calculating the sucrose consumption during the experiment for 35 consecutive days.

### 2.6. Immunohistochemistry of Corticotrophin-Releasing Factor (CRF) and Tyrosine Hydroxylase (TH)

For the immunohistochemical procedures, rats were randomly chosen from each of the five experimental group of rats used in the several behavioral tests. For immunohistochemical studies, the three animals in each group were deeply anesthetized with sodium pentobarbital (80 mg/kg, by intraperitoneal injection) and perfused through the ascending aorta with normal saline (0.9%) followed by 300 mL (per rat) of 4% paraformaldehyde in 0.1 M phosphate-buffered saline (PBS). The brains were removed in a randomized order, postfixed overnight, and cryoprotected with 20% sucrose in 0.1 M PBS at 4°C. Coronal sections 30 *μ*m thick were cut through the hypothalamus and locus coeruleus (LC) using a cryostat (Leica CM1850; Leica Microsystems Ltd., Nussloch, Germany). The sections were immunostained for CRF and TH expression using the avidin-biotin-peroxidase complex (ABC) method. Briefly, the sections were incubated with primary goat anti-CRF antibody (1 : 500 dilution; Santa Cruz Biotechnology Inc., California, CA, USA) and sheep anti-TH antibody (1 : 2000 dilution; Chemicon International Inc., Temecular, CA, USA) in PBST (PBS plus 0.3% Triton X-100) for 72 h at 4°C. The sections were incubated for 120 min at room temperature with secondary antibody. The secondary antibodies were obtained from Vector Laboratories Co. (Burlingame, CA, USA) and diluted to 1 : 200 in PBST containing 2% normal serum. To visualize immunoreactivity, the sections were incubated for 90 min in ABC reagent (Vectastain Elite ABC kit; Vector Labs. Co., Burlingame, CA, USA) and incubated in a solution containing 3,3′-diaminobenzidine (DAB; Sigma-Aldrich Chemical Co., St. Louis, MO, USA). Finally, the tissues were washed in PBS, followed by a brief rinse in distilled water, and mounted individually onto slides. Images were captured using the AxioVision 3.0 imaging system (Carl Zeiss, Inc., Oberkochen, Germany) and processed using Adobe Photoshop (Adobe Systems, Inc., San Jose, CA, USA). The sections were viewed at 100 × magnification, and the numbers of cells within 200 × 200 *μ*m^2^ grids were counted by observers blinded to the experimental groups. Counting immune-positive cells was performed in at least three different hypothalamus or LC sections per rat brain. The cells were obtained according to the stereotactic atlas of Paxinos and Watson [27].

### 2.7. Total RNA Preparation and RT-PCR Analysis

The expression levels of BDNF mRNA were determined by reverse transcription-polymerase chain reaction (RT-PCR). The brain hippocampus was isolated from three rats per group. After decapitation, the brain was quickly removed and stored at −80°C until use. The total RNA was prepared from the brain tissue using a TRIzol reagent (Invitrogen Co., Carlsbad, CA, USA) according to the supplier's instructions. Complementary DNA was first synthesized from total RNA using reverse transcriptase (Takara Co., Shiga, Japan). PCR was performed using a PTC-100 programmable thermal controller (MJ Research, Inc., Watertown, MA, USA). The operating conditions were as follows: for glyceraldehydes-3-phosphate dehydrogenase (GAPDH), 30 cycles of denaturation at 95°C for 30 sec, annealing at 58°C for 30 sec, and extension at 72°C for 30 sec; for BDNF, 27 cycles of denaturation at 95°C for 30 sec, annealing at 57°C for 30 sec, and extension at 72°C for 30 sec. All primers were designed using published mRNA sequences of those cytokines and a primer designing software, Primer 3, offered by the Whitehead Institute for Biomedical Research (Cambridge, MA, USA; http://primer3.wi.mit.edu). The following sequences were used: for GAPDH (409 bp), (forward) 5′-ATC CCA TCA CCA TCT TCC AG-3′ and (reverse) 5′-CCT GCT TCA CCA CCT TCT TG-3′; for BDNF (153 bp), (forward) 5′-CAG GGG CAT AGA CAA AAG-3′ and (reverse) 5′-CTT CCC CTT TTA ATG GTC-3′. The PCR products were separated on 1.2% agarose gels and stained with ethidium bromide. The density of each band was quantified using an image-analyzing system (i-Max, CoreBio System Co., Seoul, Republic of Korea). The expression levels were compared with each other by calculating the relative density of the target band, such as BDNF, to that of GAPDH.

### 2.8. Statistical Analysis

All measurements were performed by an independent investigator blinded to the experimental conditions. The results in the figures are expressed as the mean ± standard error of the means (SE). Statistical analysis was performed using one- or two-way analysis of variance (ANOVA) with independent and repeated measures in accordance with the experimental design. Between subjects one- or two-way ANOVA was used to analyze the effect of morphine treatment and time in time-course experiments, or morphine and acupuncture treatments. In case of significant main effect or interactions following ANOVA, multiple group comparisons were performed using Tukey's post hoc analysis. Statistical significance was defined as *P* < 0.05.

## 3. Results

### 3.1. Experiment 1

#### 3.1.1. Body Weight and Consumed Sucrose Intake in Morphine Dependence

**Figure 2:**
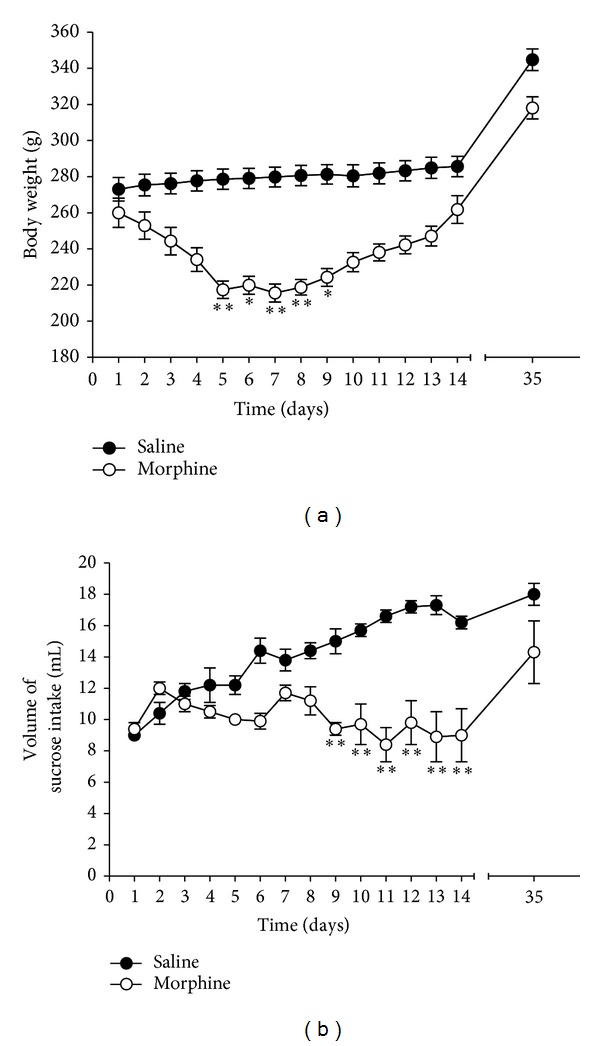
Body weight (a) and consumed sucrose intake (b) in repeated morphine administration. The experimental groups included saline-treated controls (Saline group, *n* = 4) and morphine-treated rats (Morphine group, *n* = 4). Data were analyzed using Student's *t*-test, with Levene's test to examine assumptions of equality of variance. *P < 0.05, **P < 0.01 versus Saline group. Vertical bars indicate SE.

Rats exposed to the repeated administration of morphine begin to exhibit decreased body weight on the first day of morphine injections. This initial reduction of body weight loss was maintained, or even exacerbated in some cases, for a sustained period of time without restoration [3]. In the present study, the body weight curve was examined daily for 35 days to identify whether the repeated administration of morphine (Morphine group) would result in body weight loss (Figure 2(a)). There was a significant decrease as of the second day of injections, which persisted for 8 days following the final morphine injection. Analysis of the body weight values revealed a significant gradual reduction of body weight gain over 35 days in the Morphine group compared to the saline-treated controls (*P* < 0.05 or *P* < 0.01). However, after 4 weeks of abstinence, morphine-treated rats recovered body weight and were similar to saline-treated rats.

To further investigate the impact of morphine dependence, a sucrose intake curve was examined daily for 35 days to identify whether the repeated administration of morphine would result in differences in the consumption of a sucrose solution relative to Saline group. There was a significant decrease, which persisted for 14 days after the final morphine injection (Figure 2(b)). Analysis of the sucrose intake values revealed a significant gradual reduction in consumed sucrose intake for 35 days in the Morphine group compared to the saline-treated controls (*P* < 0.01). However, after 4 weeks of abstinence, morphine-treated rats recovered sucrose consumption and were similar to saline-treated rats.

#### 3.1.2. Locomotor Activity in Morphine Dependence

**Figure 3:**
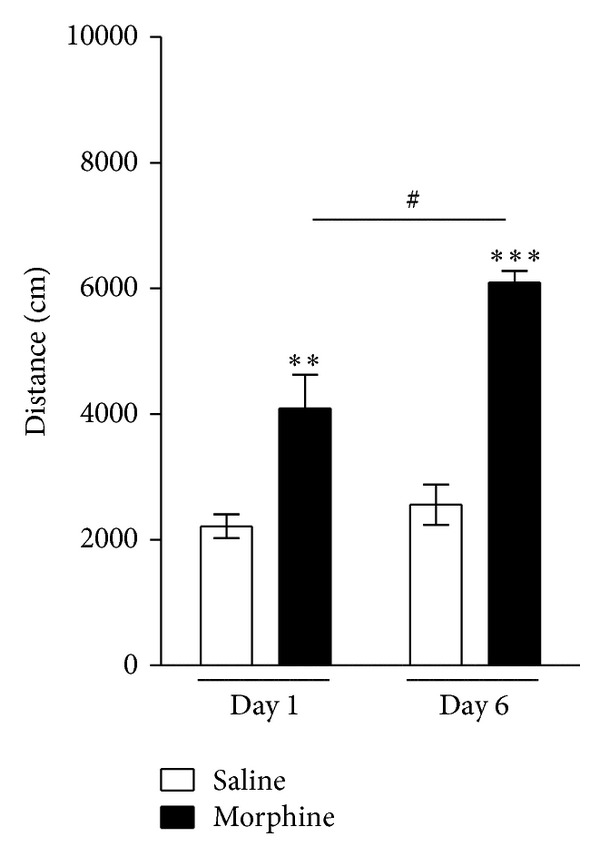
Locomotor activity during repeated morphine administration in the development phase over 6 days. The experimental groups included saline-treated controls (Saline group, *n* = 4) and morphine-treated rats (Morphine group, *n* = 4). Data were analyzed using a two-way ANOVA followed by Tukey's post hoc test. **P < 0.01, ***P < 0.001 versus Saline group; ^#^ *P* < 0.05 versus 1-week time point in the Morphine group. Vertical bars indicate SE.

To verify the development of morphine dependence induced by the repeated administration of morphine over 5 consecutive days, locomotor activity was compared between saline-treated rats and morphine-treated rats (Figure 3). Two-way ANOVA performed on the activity scores following the drug injections indicated a significant effect of groups difference (*F*(1, 28) = 27.390, *P* < 0.001), effect of days (*F*(3, 28) = 488.961, *P* < 0.001), and groups × days interactions (*F*(3, 28) = 49.145, *P* < 0.001). During development of morphine dependence, the locomotor activity in the Morphine group was significantly greater than that in the Saline group (*P* < 0.01 or *P* < 0.001). There was also a significant increase in locomotor activity in the Morphine group on day 6 compared to day 1, reflecting the development of morphine dependence (*P* < 0.05). This finding of morphine-induced changes in locomotor activity was consistent with the finding of our previous studies [28].

#### 3.1.3. Physical Dependence during Protracted Abstinence following Repeated Morphine Administration

**Figure 4:**
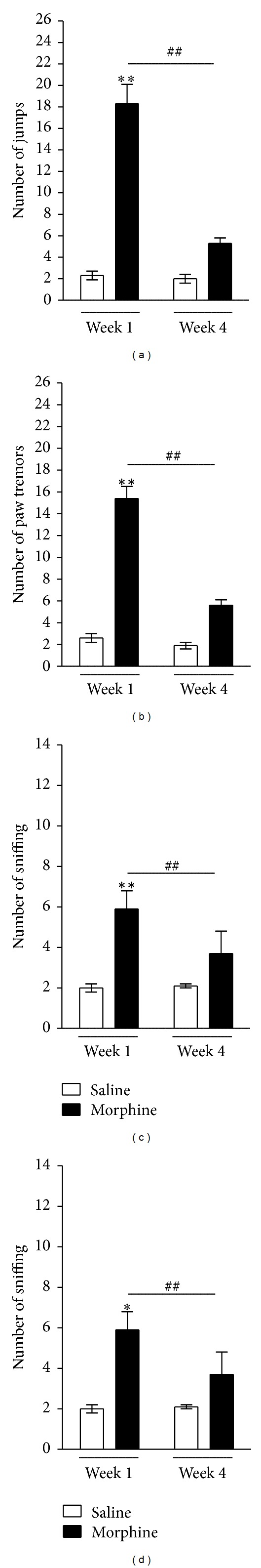
Jumps (a), paw tremors (b), shakes (c), and sniffing (d) were indicators of physical dependence at 1 or 4 weeks of protracted abstinence following repeated morphine administration. The experimental groups included saline-treated controls (Saline group, *n* = 4) and morphine-treated rats (Morphine group, *n* = 4). Data were analyzed using a two-way ANOVA followed by Tukey's post hoc test. *P < 0.05, **P < 0.01 versus Saline group; ^#^ *P* < 0.05, ^##^ *P* < 0.01 versus 1-week time point in the Morphine group. Vertical bars indicate SE.

The establishment of physical dependence following chronic morphine exposure was verified by measuring naloxone-precipitated withdrawal symptoms (Figure 4). Two hours after the final morphine injection on day 6, a single naloxone was administered. Morphine-treated rats showed significantly more withdrawal signs in response to naloxone such as jumps, paw tremors, shakes, and sniffing compared to the saline-treated controls (*P* < 0.05 or *P* < 0.01 for all parameters). Two-way ANOVA performed on the jumps scores following the drug injections indicated a significant effect of groups difference (*F*(1,28) = 59.785, *P* < 0.001), effect of days (*F*(3,28) = 196.005, *P* < 0.001), and groups × days interactions (*F*(3,28) = 47.456, *P* < 0.001). Morphine-abstinent rats jumped significantly more than saline-treated rats after 1 week of abstinence (*P* < 0.01; Figure 4(a)), but this difference was no longer significant after 4 weeks of abstinence compared to the 1 week of abstinence (*P* < 0.01). Likewise, morphine-abstinent rats exhibited significantly more paw trembling and shaking than saline-treated rats after 1 week of abstinence (*P* < 0.01; Figures 4(b) and 4(c)), but this difference was no longer significant after 4 weeks of abstinence compared to 1 week of abstinence (*P* < 0.01). Morphine-abstinent rats also sniffed significantly more than saline-treated rats after 1 week of abstinence (*P* < 0.05; Figure 4(d)), but this difference was no longer significant after 4 weeks of abstinence compared to the 1 week of abstinence (*P* < 0.05). Thus, jumping behavior, paw tremors, shaking, and sniffing decreased with the progression of abstinence, indicating that physical dependence attenuates as abstinence unfolds. This is consistent with previous studies [3].

#### 3.1.4. Despair- and Anxiety-Like Behaviors during Protracted Abstinence following Repeated Morphine Administration

**Figure 5:**
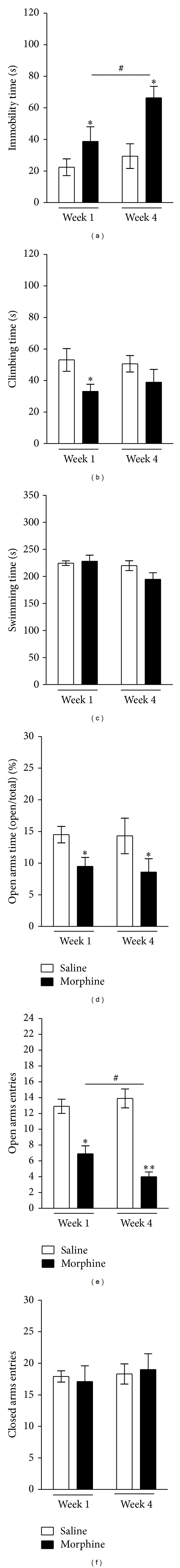
Changes in immobility time (a), climbing time (b), and swimming time (c) in the FST and the percentage of time spent in open-arm exploration (d) and the number of entries into open arms (e) and closed arms (f) in the EPM test at 1 or 4 weeks of protracted abstinence following repeated morphine administration. Data were analyzed using a two-way ANOVA followed by Tukey's post hoc test. *P < 0.05, **P < 0.01 versus Saline group; ^#^ *P* < 0.05 versus 1-week time point in the Morphine group. Vertical bars indicate SE.

During withdrawal for either 1 or 4 weeks following repeated exposure to chronic morphine, morphine-treated rats exhibit a significant depression-like phenotype or despair-like responses, characterized by an increased duration of immobility and decreased time of climbing behavior during the FST compared to the saline-treated controls (Figures 5(a) and 5(b)). Rats were subjected to the FST either 1 or 4 weeks after the final injection of morphine. Two-way ANOVA performed on the immobility scores following the drug injections indicated a significant effect of groups difference (*F*(1,28) = 6.525, *P* < 0.01), effect of days (*F*(3,28) = 107.855, *P* < 0.001), and groups × days interactions (*F*(3,28) = 23.745, *P* < 0.001). Immediately after the final morphine administration (1-week abstinence period), a significant increase in immobility time and a significant decrease in climbing behavior were observed in the Morphine group during 5 min in the FST relative to the Saline group (*P* < 0.05). Furthermore, at 4 weeks after withdrawal from morphine administration, morphine-treated rats showed a significant increase in durations of immobility time during the FST compared to 1 week of abstinence (*P* < 0.05). Therefore, despair-like behavior was enhanced following exposure to chronic morphine, and, importantly, these effects were strong only after a prolonged abstinence period. However, withdrawal from repeated morphine exposure did not lead to significant differences in swimming behavior among the groups and had no influence on ambulatory activity in the FST (*P* = 0.227; Figure 5(c)). Following withdrawal from morphine administration, the depressive symptoms persisted for 4 weeks (i.e., increased immobility without any effect on ambulatory activity).

Anxiety expressed as a decrease in open-arm exploration in the EPM test was also analyzed. Rats were challenged in the EPM test either 1 or 4 weeks after the final injection of morphine (Figures 5(d) and 5(e)). Two-way ANOVA performed on the open-arm exploration scores following the drug injections indicated a significant effect of groups difference (*F*(1,28) = 2.424, *P* < 0.05), effect of days (*F*(3,28) = 140.035, *P* < 0.001), and groups × days interactions (*F*(3,28) = 34.745, *P* < 0.001). Immediately after the final morphine administration (1-week abstinence period), a significant decrease in both the percentage of time spent and the number of entries into open arms was observed in the Morphine group compared to the Saline group (*P* < 0.05 or *P* < 0.01). Furthermore, at 4 weeks after withdrawal from morphine administration, morphine-treated rats significantly decreased the number of entries in the arena, which decreased open-arm exploration during the EPM test relative to morphine-treated rats at 1 week following withdrawal from morphine (*P* < 0.05). Therefore, anxiety behavior was increased following exposure to chronic morphine, and, importantly, these effects were strong only after a prolonged abstinence period. Conversely, withdrawal from repeated morphine exposure did not lead to significant differences in the number of entries into closed arms among the groups and had no influence on ambulatory activity in the EPM test (*P* = 0.308; Figure 5(f)). Following withdrawal from morphine administration, the anxiety-like symptoms persisted for 4 weeks.

#### 3.1.5. Aberrant Social Behaviors during Protracted Abstinence following Repeated Morphine Administration

**Figure 6:**
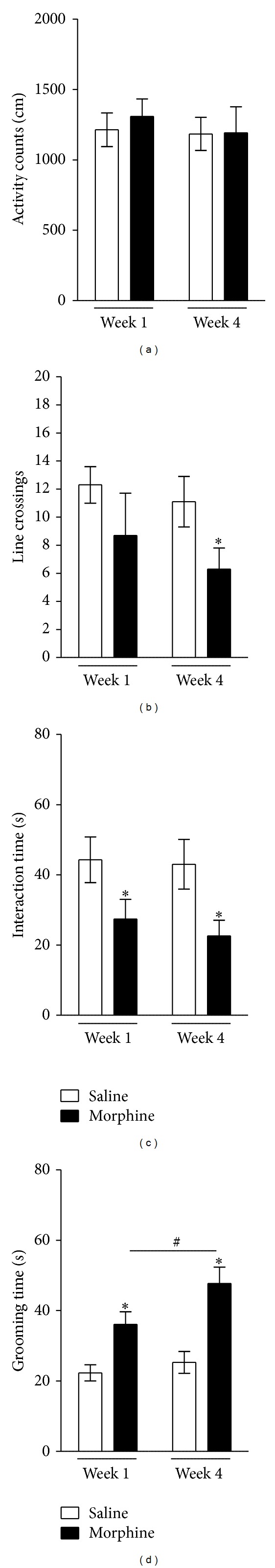
Changes in activity counts (a) and line crossing (b) in the OFT and the interaction time (c) and grooming time (d) in the social interaction test at 1 or 4 weeks of protracted abstinence following repeated morphine administration. Data were analyzed using a two-way ANOVA followed by Tukey's post hoc test. *P < 0.05 versus Saline group; ^#^ *P* < 0.05 versus 1-week time point in the Morphine group. Vertical bars indicate SE.

Reduced exploratory behavior expressed as a decrease in locomotor activity or total number of line crossings in the OFT was also analyzed. Rats were challenged in the OFT either 1 or 4 weeks after the final injection of morphine (Figures 6(a) and 6(b)). Two-way ANOVA performed on the locomotor activity scores following the drug injections indicated a significant effect of groups difference (*F*(1,28) = 0.171, *P* = 0.915), effect of days (*F*(3,28) = 308.276, *P* < 0.001), and groups × days interactions (*F*(3,28) = 45.475, *P* = 0.457). There were no differences in total distance travelled in the arena in the OFT between the groups (*P* = 0.220). However, the Morphine group displayed a significant decrease in the total number of line crossings in the arena at 4 weeks after the final morphine injection compared to the Saline group (*P* < 0.05).

During withdrawal for either 1 or 4 weeks following repeated exposure to chronic morphine, morphine-treated rats exhibited a markedly altered “social interaction” phenotype or anxiety-like responses, characterized by decreased total social exploration time for pairs of the same treatment group (social) and increased grooming time (individual) during the OFT compared to the saline-treated controls (Figures 6(c) and 6(d)). Immediately after the final morphine administration (1-week abstinence period), morphine-treated rats exhibited a decrease in interaction time and an increase in grooming time compared to the Saline group (*P* < 0.05). Post hoc analysis revealed that morphine-treated pairs of rats interacted less than saline-treated pairs of rats either at 1 or 4 weeks of abstinence. At 4 weeks after withdrawal from morphine administration, rats in the Morphine group showed a significant enhancement of grooming time in the OFT compared to 1 week of abstinence (*P* < 0.05). Following withdrawal from morphine administration, the altered social interaction persisted for 4 weeks.

### 3.2. Experiment 2

#### 3.2.1. Effects of Acupuncture Stimulation of SP6 on Morphine-Induced Despair- and Anxiety-Like Behavior during Protracted Abstinence

**Figure 7:**
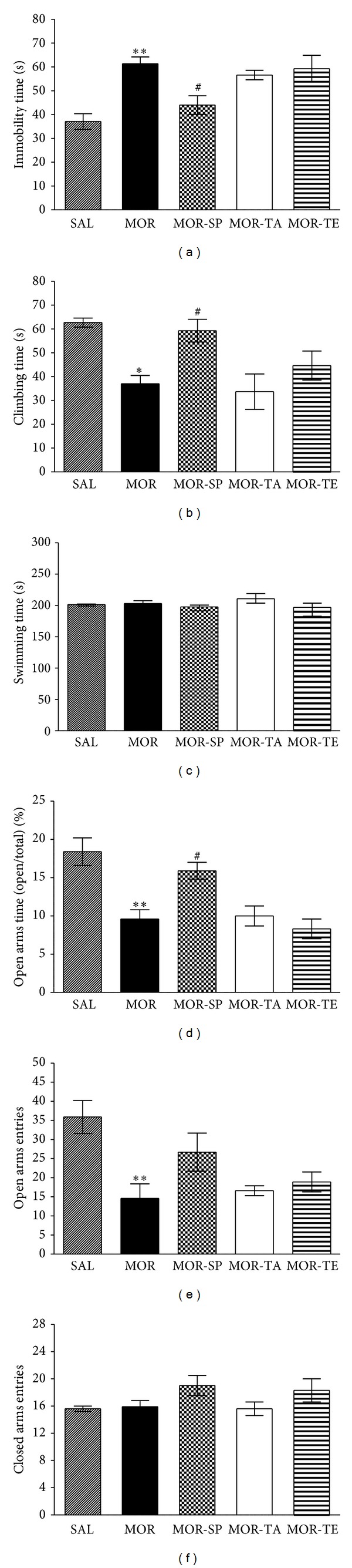
Effect of acupuncture on immobility time (a), climbing time (b), and swimming time (c) in the FST and the percentage of time spent in open-arm exploration (d) and the number of entries into open arms (e) and closed arms (f) in the EPM test at 4 weeks of protracted abstinence following repeated morphine administration. The rats were randomly divided into five groups of seven individuals each as follows. The withdrawal group following repeated morphine administration was given morphine (20, 40, 60, 80, 100 mg/kg-body weight, *s.c.*, MOR group, *n* = 7) twice a day for 5 consecutive days. One or 4 weeks following the final morphine injection, behavioral responses were tested during this period. The vehicle-treated rats (as a negative control in the development of addiction withdrawal model) were administered saline (0.9% NaCl, *s.c.*) instead of morphine in the same way (SAL group, *n* = 7). The acupuncture-treated groups were divided as follows: Sanyinjiao (SP6) acupoint-stimulated plus morphine-treated group (MOR-SP, *n* = 7), Waiguan (TE5) acupoint-stimulated plus morphine-treated group (MOR-TE, *n* = 7), and nonacupoint (on the tail)-stimulated plus morphine-treated group (MOR-TA, *n* = 7). Data were analyzed using a one-way ANOVA followed by Tukey's post hoc test. *P < 0.05, **P < 0.01 versus SAL group; ^#^ *P* < 0.05 versus MOR group. Vertical bars indicate SE.

The effects of acupuncture stimulation at SP6 during protracted abstinence were evaluated during the abstinence period using the FST. In accordance with previous results, rats in the MOR group showed greater immobility than did those in the saline-treated control rats (SAL group). Acupuncture abolished this difference, and thus rats in the MOR-SP group had significantly reduced immobility during 5 min in the FST compared to the MOR group at 4 weeks after the final morphine injection (*P* < 0.05; Figure 7(a)). This finding suggests that acupuncture stimulation of SP6, but not of TE5 or the tail, significantly restores depression-like despair behavior. Another key behavior, climbing behavior was also analyzed [29]. Morphine-treated rats were less active compared to the saline-treated control rats, and acupuncture treatment eliminated this difference. Rats in the MOR-SP group showed significantly restored climbing behavior during 5 min in the FST compared to the MOR group (*P* < 0.05; Figure 7(b)). This finding indicates that acupuncture stimulation of SP6 during the 4-week abstinence period is able to prevent the development of despair-like behaviors. Withdrawal from repeated morphine exposure did not lead to significant differences in swimming behaviors among the groups in the FST (*P* = 0.245; Figure 7(c)).

The effects of acupuncture stimulation of SP6 during protracted abstinence were also evaluated during the abstinence period using the EPM test. Rats in the MOR-SP group spent significantly more time into the open arms of the maze compared to the MOR group at 4 weeks after the final morphine injection (*P* < 0.05; Figure 7(d)). Similarly, rats in the MOR-SP group exhibited a slightly increased number of entries into the open arms of the maze, which was formerly decreased by withdrawal from repeated morphine compared to the MOR group (*P* = 0.105; Figure 7(e)). These findings indicate that acupuncture stimulation of SP6, but not of TE5 or the tail, during protracted abstinence significantly ameliorates anxiety-like behavior. The absence of significant differences in the number of closed-arm entries among the groups in the EPM test suggests that this anxiety-like behavior in morphine-treated rats cannot be attributed to differences in locomotor activity (*P* = 0.229; Figure 7(f)). This finding suggests that acupuncture stimulation of SP6 during the 4-week abstinence period is able to prevent the development of anxiety-like behavior.

#### 3.2.2. Effects of Acupuncture Stimulation of SP6 on Morphine-Induced Aberrant Social Behaviors during Protracted Abstinence

**Figure 8:**
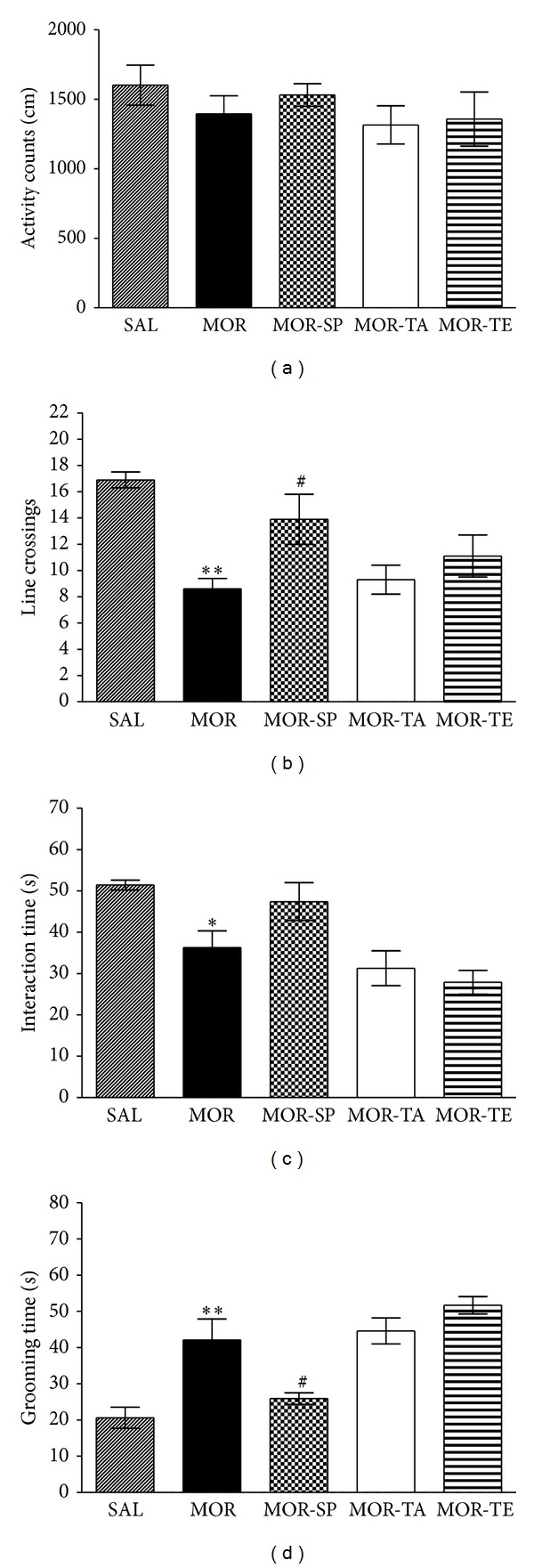
Effect of acupuncture on activity counts (a) and line crossing (b) in the OFT and the interaction time (c) and grooming time (d) in the social interaction test at 4 weeks of protracted abstinence following repeated morphine administration. Data were analyzed using a one-way ANOVA followed by Tukey's post hoc test. *P < 0.05, **P < 0.01 versus SAL group; ^#^ *P* < 0.05 versus MOR group. Vertical bars indicate SE.

The effects of acupuncture stimulation of SP6 during protracted abstinence were evaluated during the abstinence period using the OFT. Parametric one-way analysis of variance ANOVA did not show any significant differences between groups in terms of total distance travelled in the OFT (*P* = 0.308; Figure 8(a)). However, rats in the MOR-SP group displayed a significant increase in total number of line crossings compared to the MOR group at 4 weeks after the final morphine injection (*P* < 0.05; Figure 8(b)). This finding indicates that acupuncture stimulation of SP6, but not of TE5 or the tail, increased exploratory behavior. It also indicates that acupuncture stimulation of SP6 during the 4-week abstinence period is able to development of exploratory behavior.

The effects of acupuncture stimulation of SP6 during protracted abstinence in rats previously exposed to morphine were then examined using social and individual parameters in the social interaction test in an open field apparatus. Rats in the MOR-SP group exhibited slightly increased interaction time in the open-field apparatus, which was formerly decreased by withdrawal from repeated morphine compared to the MOR group (*P* = 0.257; Figure 8(c)). However, rats in the MOR-SP group also spent significantly less time grooming in the open field apparatus compared to the MOR group (*P* < 0.05; Figure 8(d)). These findings indicate that acupuncture stimulation of SP6 during the 4-week abstinence period prevented the development of aberrant social behaviors.

#### 3.2.3. Effects of Acupuncture Stimulation of SP6 on Morphine-Induced CRF- and TH-Like Immunoreactivities during Protracted Abstinence

**Figure 9:**
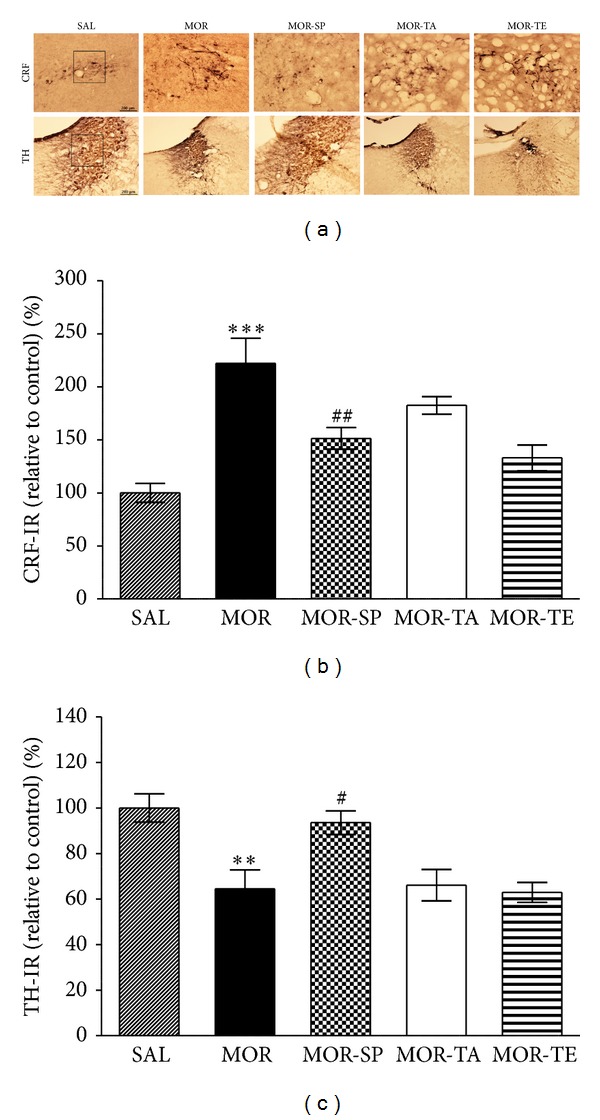
Representative photomicrographs showing CRF expression in the paraventricular nucleus (PVN) of the hypothalamus and TH expression in the locus coeruleus (LC) (a). Effect of acupuncture on the expression of CRF (b) and TH (c) at 4 weeks of protracted abstinence following repeated morphine administration. The scale bar represents 100 *μ*m. **P < 0.01, ***P < 0.001 versus SAL; ^#^ *P* < 0.05, ^##^ *P* < 0.01 versus MOR group.

Many studies have shown that CRF levels are correlated with anxiety in animal models of depression, suggesting a possible link between high level expression of CRF and predisposition to anxiety or morphine withdrawal-induced depression [30]. In addition, previous studies suggested that morphine dependence and withdrawal induce hyperactivity of noradrenergic pathways and an increase in TH modulation in the LC [31]. It has been proposed that clinical anxiety or depression may be the result of alterations in the activity of the LC in central noradrenergic system [32]. Following withdrawal from repeated morphine injections, CRF-like immunoreactivity was primarily detected in the cell bodies of various hypothalamic regions, including the paraventricular nucleus (PVN; Figure 9(a)). In the brains of the MOR group, the number of CRF immunoreactive neurons in the PVN was increased by 222.08%. Analysis of the numbers of CRF-immunoreactive neurons values revealed a significant increase in CRF expression in the MOR group compared to the SAL group (*P* < 0.001; Figure 9(b)). The number of CRF-immunoreactive neurons was significantly decreased in the hypothalamic PVN region of the MOR-SP group compared to the MOR group (*P* < 0.01). TH-like immunoreactivity was also analyzed in the cell bodies of major noradrenergic regions, including the LC (Figure 9(a)). In the brains of the MOR group, the number of TH immunoreactive neurons in the LC was decreased by 64.52%. Analysis of the numbers of TH-immunoreactive neurons values revealed that rats repeatedly exposed to morphine exhibit a significant decrease of TH expression compared to the SAL group (*P* < 0.01; Figure 9(c)). The number of TH-immunoreactive neurons was significantly increased in the central adrenergic regions of the MOR-SP group compared to the MOR group (*P* < 0.05). This finding indicates that the increase in the number of CRF-immunoreactivity and decrease in the number of TH-immunoreactivity induced by withdrawal were significantly restored by acupuncture stimulation to the SP6 acupoint.

#### 3.2.4. Effects of Acupuncture Stimulation of SP6 on Morphine-Induced BDNF mRNA Expression in the Hippocampus

**Figure 10:**
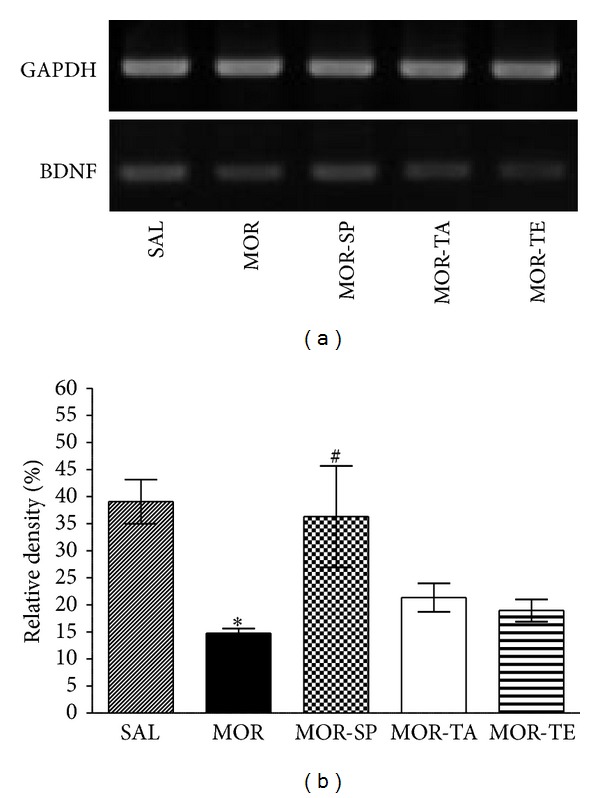
RT-PCR bands (a) and their relative intensities (b) of brain-derived neurotrophic factor (BDNF) mRNA in the rat hippocampus at 4 weeks of protracted abstinence following repeated morphine administration. *P < 0.05 versus SAL group; ^#^ *P* < 0.05 versus MOR group.

BDNF may drive synaptic changes underlying drug craving and drug-seeking behavior [33]. The effects of acupuncture stimulation to the SP6 acupoint on BDNF mRNA expression were investigated in the rat hippocampus following withdrawal from repeated morphine injection using RT-PCR analysis (Figure 10). BDNF mRNA expression was normalized against GAPDH mRNA, a housekeeping gene used as an internal control. BDNF mRNA expression in the hippocampus of the MOR group was significantly decreased compared to the SAL group (*P* < 0.05). The decreased BDNF mRNA expression in the MOR groups was significantly restored in the MOR-SP group (*P* < 0.05).

## 4. Discussion

The present results demonstrate that protracted abstinence following repeated morphine administration increased despair-related, anxiety-like, and aberrant social behaviors in rats. However, these results clearly establish that acupuncture stimulation of SP6 during protracted morphine abstinence for 4 weeks significantly decreased the duration of immobility in the FST, increased open-arm exploration in the EPM test, and reversed increases in aberrant social behaviors by modulating hypothalamic CRF and the noradrenergic system in the CNS. Moreover, in the present study, there was a decrease in the expression of BDNF mRNA in the rat hippocampus as well as an increase in depression- and anxiety-like symptoms following protracted abstinence from repeated morphine administration. However, the acupuncture stimulation of SP6 during the protracted abstinence period restored BDNF mRNA levels. Thus, the results of this study support the possibility that acupuncture might have antidepressant and antianxiolytic effects.

Morphine abuse continues to be a serious risk factor for various problems in society and may induce depressive symptoms. Conversely, depressed mood may contribute to drug abuse in accord with a self-medication hypothesis [34]. For the past several decades, several antidepressants have been implicated in the alleviation of both types of illnesses such as depression and drug abuse [5, 6]. Therefore, there is a need to develop effective medications that can treat morphine withdrawal symptoms, particularly with regard to relapse into drug-seeking behavior [35]. In the current model of morphine abstinence, abstinent rats develop depressive-like deficits, which increased with the duration of abstinence, while sensitivity to the symptoms of naloxone-precipitated withdrawal declines, as described previously in other studies [36]. Protracted abstinence from repeated morphine administration elicits despair-related, anxiety-like, and aberrant social behaviors in the rats, as assessed by the FST [2], the EPM [37], and the social interaction test [38]. In the present study, increases in immobility in the FST and decreases in open-arm exploration time in the EPM test appeared as a trend after 1 week of abstinence and became significant after 4 weeks. Thus, changes in depression- and anxiety-like behaviors may be observed 4 weeks after abstinence.

Furthermore, morphine dependence induced by repeated administration of morphine over 5 days can affect the consumption of a sucrose solution, and this may be closely correlated with the progression or exacerbation of motivation of drug reinforcement. Many studies have shown that a higher dose of morphine reduced intake of the sucrose in free-feeding rats [39]. Also, the morphine dependence greatly reduced intake of the saccharin or reduced the motivation to acquire the sucrose reinforcement when assessed in food-deprived rats using a saccharin conditioned stimulus [39]. Impulsive choice or impulsivity can be defined as an exaggerated preference for small immediate rewards and can be measured in humans and animals using a discounting of morphine dependence. We observed that changes in sucrose consumption in dependent rats mirror changes in impulsivity. Dependent rats show both increased impulsivity and decreased sucrose consumption during dependence, but not after abstinence [40].

The observed immobility behavior by rats in the FST is analogous to a state of lowered mood or helplessness and depression in humans [41]. Although the FST provides information about mood (i.e., depression and anxiety) in rodents, it is important to use caution when extrapolating the data to humans. In inescapable situations, immobility reflects a passive coping strategy associated with resignation, which is a behavioral measure of emotional despair [41]. The current results are consistent with previous findings showing that behavior related to withdrawal from repeated morphine administration includes increased immobility during the FST [42]. Acupuncture stimulation of SP6 significantly decreased immobility and increased climbing behavior in the FST. There was no effect on swimming time in the FST, which confirms that the antidepressant-like activity was not caused by a motor function deficit [30].

Anxiety is another complex feature of depression, and anxiety-like symptoms in chronically stressed animals are not surprising [30]. Many studies have suggested that rats receiving repeated morphine administration exhibit decreases in the proportion of time spent and the number of entries into the open arms of the EPM test [3, 43]. It should be noted that a heightened anxiety level was observed in morphine-treated rats at 4 weeks, and this anxiogenic-like effect influenced social and despair behaviors, as morphine-treated rats showed similar social interaction times and immobility in the FST [44]. However, acupuncture stimulation of SP6 during protracted abstinence for 4 weeks significantly reduced anxiety-like behaviors in the EPM test, as indicated by an increase in the percentage of time spent into the open arms. Accordingly, these results suggest that chronic acupuncture stimulation of SP6 ameliorates anxiety-related behaviors that persist for 4 weeks in rats after withdrawal from repeated morphine administration.

Concurrently, low sociability, as reflected by decreased total social exploration time between pairs of rats, became evident during the protracted abstinence period [3]. At 4 weeks, rats also exhibited a strong increase in self-grooming behavior, which was executed in a stereotyped and incomplete sequence that could reflect displacement behavior, a compulsive-type behavior to avoid coping [45]. Thus, the current results indicate the emergence of gradual social withdrawal that parallels the development of despair behaviors in rats experiencing protracted morphine abstinence [3]. Alternatively, decreased social interaction may also result from increased levels of anxiety, but this seems unlikely under the current experimental conditions [46]. Low light and familiar conditions that are not expected to generate anxiety were deliberately used. Overall, acupuncture stimulation of SP6 during protracted abstinence for 4 weeks was able to prevent the development of aberrant social behaviors.

Previous studies have shown that increased CRF activity in the brain is associated with behavioral and physiological manifestations of drug withdrawal and relapses in drug-taking behaviors in both animals and human clinical populations [30, 37]. CRF contributes to the anxiogenesis and aversive symptoms of withdrawal from several drugs of abuse, including morphine [47]. The present data suggest that CRF circuits in the hypothalamus were potently activated by morphine administration, and this activation might be responsible for inducing depression and anxiety related to morphine withdrawal [48]. Our results show that acupuncture stimulation significantly blocked the withdrawal-induced increase in CRF immunoreactivity in the PVN, suggesting that the antidepressive effect of acupuncture stimulation is closely associated with CRF modultiaon in the PVN. Furthermore, alterations in hypothalamic CRF activity underlie the antidepressant and anxiolytic activities of acupuncture stimulation of SP6 following protracted abstinence from repeated morphine administration in rats.

Furthermore, many studies have shown that withdrawal from morphine leads to extreme anxiety, which is accompanied by several physical disturbances, most of which are linked to the activation of brainstem regions such as the LC [32]. TH is an enzyme involved in the response to morphine withdrawal-related psychopathological conditions, such as depression and anxiety [31]. Accordingly, TH expression in the LC is decreased following repeated morphine exposure and subsequent withdrawal, possibly due to long-term drug seeking or relapse in anticipation of drug discontinuation [49]. Therefore, the current results are consistent with previous reports indicating that depression- and anxiety-like behaviors induced by morphine withdrawal are the result of alterations in the central noradrenergic system [32, 50]. TH dysfunction evolves after cessation of drug exposure and persists for an extended period of time during protracted abstinence [12, 51]. We demonstrated that acupuncture stimulation significantly increased TH-like immunoreactivity in the LC of rats subjected to protracted abstinence following chronic morphine exposure. Therefore, these findings may help to explain how acupuncture stimulation at SP6 may affect the behavioral signals induced by reduced TH expression in the LC, thereby normalizing behavioral and neurochemical responses. Importantly, the effects of acupuncture stimulation may be relatively specific to particular acupoints, demonstrating that treatment with acupuncture therapy specifically influences long-term neural adaptations [52]. Such adaptations may, in turn, restore functional alterations in the noradrenergic system and reverse behavioral depressive-like symptoms [53]. The present findings strongly support the notion that noradrenergic functioning is altered in protracted abstinence from morphine. Thus, acupuncture stimulation of SP6 is capable of attenuating complex behaviors associated with depression and anxiety via the modulation of the central noradrenergic system.

Other findings have suggested that increased BDNF expression in the hippocampus produces long-lasting enhancement of cocaine seeking and locomotor stimulation during repeated cocaine administration [54, 55]. Moreover, BDNF is a central neurotrophic factor related to critical CNS functions and is important in the control of mood, emotion, and cognition [56]. Rats experiencing withdrawal from morphine administration exhibited a significant reduction in BDNF levels, as in previous studies, and acupuncture stimulation at SP6 prevented this reduction. In the present study, acupuncture stimulation of SP6 restored the decreased expression level of BDNF mRNA in the hippocampus and ameliorated depression- and anxiety-like behaviors in rats experiencing protracted abstinence from morphine administration.

Interestingly, 5 minutes of acupuncture stimulation was enough to modulate the behavioral and neurochemical responses induced by withdrawal. Additionally, only acupuncture stimulation to the SP6 acupoint elicited significant responses, in contrast to an acupoint on a different meridian, TE5, or a nonacupoint on the tail. SP6 has been used clinically to treat mental and psychosomatic disorders and is known to produce a sedative, tranquilizing, and antiemetic effect under a variety of stresses [14]. These results suggest that stimulation of the acupuncture point spreads throughout the body at a rapid rate and its effect is highly point specific, at least for modulating the depression- and anxiety-like symptoms associated with morphine discontinuation [12, 57]. Based on these results, it is suggested that acupuncture stimulation of SP6 during protracted abstinence may inhibit the development of morphine dependence, and acupuncture therapy can be a powerful regulator of psychiatric disorders as well as a potential therapy against disease.

Acupuncture treatment is performed by inserting needles into the subject at specific points and manipulating them [19]. It improves reversible malfunctions of the body via direct activation of various brain pathways and thus contributes to the restoration of normal systemic balance and may treat mental dysfunctions involving drug abuse [58]. Although acupuncture stimulation is known to be clinically effective for alleviating psychostimulant-induced withdrawal symptoms, the underlying mechanisms of acupuncture stimulation in those therapies have not been investigated. Currently, acupuncture is a relevant therapy in complementary and alternative medicine for managing various psychosomatic diseases such as drug abuse, stress, depression, and anxiety [12, 59, 60].

The neural mechanisms underlying the influence of acupuncture on psychological distress have yet to be clarified. Acupuncture may exert its effects by influencing central noradrenergic pathways which support emotional states. Indeed, acupuncture stimulation leads to inhibition of CRF and TH, which are implicated in the etiology of depression. Furthermore, acupuncture may also stimulate sensory nerves, induce the release of endogenous opioids, modulate autonomic nervous system activity [61] and perhaps influence other hormones that medicate mood [62]. Future studies should investigate whether applied acupuncture can also reduce peripheral stress factors. Furthermore, our study will assess whether acupuncture can also modulate cellular immune functions in the rats.

## 5. Conclusion

In summary, the current study establishes a direct connection between protracted abstinence from morphine and depressive-like symptoms in rats. These results demonstrate that acupuncture stimulation of SP6 reduces the depression- and anxiety-like symptoms strongly associated with morphine abstinence, probably by modulating hypothalamic CRF and the noradrenergic system that underlies mood disruption. This indicates that acupuncture stimulation of SP6 might be effective in preventing patients with drug addiction from relapsing into drug seeking while trying to quit, by relieving some of the discomfort of morphine withdrawal symptoms including depression and anxiety. Therefore, acupuncture stimulation may be a useful therapy in the development of alternative medicines for treating morphine withdrawal-related symptoms, such as depression and anxiety.

## References

[1] Zhou W, Zhang F, Liu H (2009). Effects of training and withdrawal periods on heroin seeking induced by conditioned cue in an animal of model of relapse. *Psychopharmacology*.

[2] Anraku T, Ikegaya N, Matsuki N, Nishiyama N (2001). Withdrawal from chronic morphine administration causes prolonged enhancement of immobility in rat forced swimming test. *Psychopharmacology*.

[3] Goeldner C, Lutz P-E, Darcq E (2011). Impaired emotional-like behavior and serotonergic function during protracted abstinence from chronic morphine. *Biological Psychiatry*.

[4] Lee B, Kim H, Shim I, Lee H, Hahm D-H (2011). Wild ginseng attenuates anxiety- and depression-like behaviors during morphine withdrawal. *Journal of Microbiology and Biotechnology*.

[5] Berton O, Nestler EJ (2006). New approaches to antidepressant drug discovery: beyond monoamines. *Nature Reviews Neuroscience*.

[6] Singh VP, Jain NK, Kulkarni SK (2003). Fluoxetine suppresses morphine tolerance and dependence: modulation of NO-cGMP/DA/serotoninergic pathways. *Methods & Findings in Experimental & Clinical Pharmacology*.

[7] Mochizuki D, Tsujita R, Yamada S (2002). Neurochemical and behavioural characterization of milnacipran, a serotonin and noradrenaline reuptake inhibitor in rats. *Psychopharmacology*.

[8] Zhao Z, Zhang H-T, Bootzin E, Millan MJ, O’Donnell JM (2009). Association of changes in norepinephrine and serotonin transporter expression with the long-term behavioral effects of antidepressant drugs. *Neuropsychopharmacology*.

[9] Kwon S, Lee B, Kim M, Lee H, Park H-J, Hahm D-H (2010). Antidepressant-like effect of the methanolic extract from Bupleurum falcatum in the tail suspension test. *Progress in Neuro-Psychopharmacology & Biological Psychiatry*.

[10] Lee JH, Kim HY, Jang EY (2011). Effect of acupuncture on naloxone-precipitated withdrawal syndrome in morphine-experienced rats: the mediation of GABA receptors. *Neuroscience Letters*.

[11] Mukaino Y, Park J, White A, Ernst E (2005). The effectiveness of acupuncture for depression—a systematic review of randomised controlled trials. *Acupuncture in Medicine*.

[12] Lee B, Shim I, Lee H-J, Yang Y, Hahm D-H (2009). Effects of acupuncture on chronic corticosterone-induced depression-like behavior and expression of neuropeptide Y in the rats. *Neuroscience Letters*.

[13] Walling A (2006). Therapeutic modulation of the psychoneuroimmune system by medical acupuncture creates enhanced feelings of well-being. *Journal of the American Academy of Nurse Practitioners*.

[14] Pavão TS, Vianna P, Pillat MM, Machado AB, Bauer ME (2010). Acupuncture is effective to attenuate stress and stimulate lymphocyte proliferation in the elderly. *Neuroscience Letters*.

[15] Park H, Yoo D, Kwon S (2012). Acupuncture stimulation at HT7 alleviates depression-induced behavioral changes via regulation of the serotonin system in the prefrontal cortex of maternally-separated rat pups. *The Journal of Physiological Sciences*.

[16] Kwon S, Kim D, Park H (2012). Prefrontal-limbic change in dopamine turnover by acupuncture in maternally separated rat pups. *Neurochemical Research*.

[17] Park H-J, Chae Y, Jang J, Shim I, Lee H, Lim S (2005). The effect of acupuncture on anxiety and neuropeptide Y expression in the basolateral amygdala of maternally separated rats. *Neuroscience Letters*.

[18] Castilho VM, Borelli KG, Brandão ML, Nobre MJ (2008). Anxiety-like symptoms induced by morphine withdrawal may be due to the sensitization of the dorsal periaqueductal grey. *Physiology & Behavior*.

[19] Lee B, Sur B-J, Kwon S (2012). Acupuncture stimulation alleviates corticosterone-induced impairments of spatial memory and cholinergic neurons in rats. *Evidence-Based Complementary and Alternative Medicine*.

[20] Huang C, Wang Y, Han J-S, Wan Y (2002). Characteristics of electroacupuncture-induced analgesia in mice: variation with strain, frequency, intensity and opioid involvement. *Brain Research*.

[21] MacPherson H, Hammerschlag R, Lewith G, Schnyer R (2008). *Acupuncture Research: Strategies for Building an Evidence Base*.

[22] Hecker HU, Steveling A, Peuker E, Kastner J (1986). *Color Atlas of Acupuncture: Body Points—Ear Points—Trigger Points*.

[23] Zhao RJ, Yoon SS, Lee BH (2006). Acupuncture normalizes the release of accumbal dopamine during the withdrawal period and after the ethanol challenge in chronic ethanol-treated rats. *Neuroscience Letters*.

[24] Pumpaisalchai W, Kaewichit S, Taesothikul T, Niwatananun W, Sanichwankul K (2005). The antidepressive effect of barakol in the forced-swimming test. *CMU Journal*.

[25] Morales-Medina JC, Dumont Y, Bonaventure P, Quirion R (2012). Chronic administration of the Y_2_ receptor antagonist, JNJ-31020028, induced anti-depressant like-behaviors in olfactory bulbectomized rat. *Neuropeptides*.

[26] Mao Q-Q, Ip S-P, Ko K-M, Tsai S-H, Che C-T (2009). Peony glycosides produce antidepressant-like action in mice exposed to chronic unpredictable mild stress: effects on hypothalamic-pituitary-adrenal function and brain-derived neurotrophic factor. *Progress in Neuro-Psychopharmacology & Biological Psychiatry*.

[27] Paxinos G, Watson C (1986). *The Rat Brain in Stereotaxic Coordinatesed*.

[28] Lee B, Kwon S, Yeom M, Shim I, Lee H, Hahm D-H (2011). Wild ginseng attenuates repeated morphine-induced behavioral sensitization in rats. *Journal of Microbiology and Biotechnology*.

[29] Vieira C, de Lima TCM, Carobrez ADP, Lino-de-Oliveira C (2008). Frequency of climbing behavior as a predictor of altered motor activity in rat forced swimming test. *Neuroscience Letters*.

[30] Lee B, Sur B, Yeom M, Shim I, Lee H, Hahm DH (2012). Effect of berberine on depression- and anxiety-like behaviors and activation of the noradrenergic system induced by development of morphine dependence in rats. *The Korean Journal of Physiology & Pharmacology*.

[31] McClung CA, Nestler EJ, Zachariou V (2005). Regulation of gene expression by chronic morphine and morphine withdrawal in the locus ceruleus and ventral tegmental area. *Journal of Neuroscience*.

[32] Núñez C, Földes A, Pérez-Flores D (2009). Elevated glucocorticoid levels are responsible for induction of tyrosine hydroxylase mRNA expression, phosphorylation, and enzyme activity in the nucleus of the solitary tract during morphine withdrawal. *Endocrinology*.

[33] Chu N-N, Zuo Y-F, Meng L, Lee DY-W, Han J-S, Cui C-L (2007). Peripheral electrical stimulation reversed the cell size reduction and increased BDNF level in the ventral tegmental area in chronic morphine-treated rats. *Brain Research*.

[34] Negus SS (2006). Choice between heroin and food in nondependent and heroin-dependent rhesus monkeys: effects of naloxone, buprenorphine, and methadone. *Journal of Pharmacology and Experimental Therapeutics*.

[35] Stewart J (2003). Stress and relapse to drug seeking: studies in laboratory animals shed light on mechanisms and sources of long-term vulnerability. *American Journal on Addictions*.

[36] Frenois F, Cador M, Caillé S, Stinus L, le Moine C (2002). Neural correlates of the motivational and somatic components of naloxone-precipitated morphine withdrawal. *European Journal of Neuroscience*.

[37] Shi J, Li S-X, Zhang X-L (2009). Time-dependent neuroendocrine alterations and drug craving during the first month of abstinence in heroin addicts. *American Journal of Drug and Alcohol Abuse*.

[38] Sukhotina IA, Bespalov AY (2000). Effects of the NMDA receptor channel blockers memantine and MRZ 2/579 on morphine withdrawal-facilitated aggression in mice. *Psychopharmacology*.

[39] Grigson PS, Lyuboslavsky PN, Tanase D, Wheeler RA (1999). Water-deprivation prevents morphine-, but not LiCl-induced, suppression of sucrose intake. *Physiology & Behavior*.

[40] Harvey-Lewis C, Perdrizet J, Franklin KB (2012). The effect of morphine dependence on impulsive choice in rats. *Psychopharmacology*.

[41] Cryan JF, Holmes A (2005). Model organisms: the ascent of mouse: advances in modelling human depression and anxiety. *Nature Reviews Drug Discovery*.

[42] Hosseinmardi N, Fathollahi Y, Naghdi N, Javan M (2009). Theta pulse stimulation: a natural stimulus pattern can trigger long-term depression but fails to reverse long-term potentiation in morphine withdrawn hippocampus area CA1. *Brain Research*.

[43] Miladi-Gorji H, Rashidy-Pour A, Fathollahi Y (2012). Anxiety profile in morphine-dependent and withdrawn rats: effect of voluntary exercise. *Physiology & Behavior*.

[44] Rygula R, Abumaria N, Flügge G, Fuchs E, Rüther E, Havemann-Reinecke U (2005). Anhedonia and motivational deficits in rats: impact of chronic social stress. *Behavioural Brain Research*.

[45] Kalueff AV, Tuohimaa P (2005). The grooming analysis algorithm discriminates between different levels of anxiety in rats: potential utility for neurobehavioural stress research. *Journal of Neuroscience Methods*.

[46] File SE, Kenny PJ, Ouagazzal A-M (1998). Bimodal modulation by nicotine of anxiety in the social interaction test: role of the dorsal hippocampus. *Behavioral Neuroscience*.

[47] Navarro-Zaragoza J, Núñez C, Laorden ML, Milanés MV (2010). Effects of corticotropin-releasing factor receptor-1 antagonists on the brain stress system responses to morphine withdrawal. *Molecular Pharmacology*.

[48] Papaleo F, Kitchener P, Contarino A (2007). Disruption of the CRF/CRF1 receptor stress system exacerbates the somatic signs of opiate withdrawal. *Neuron*.

[49] Watanabe T, Nakagawa T, Yamamoto R, Maeda A, Minami M, Satoh M (2003). Involvement of noradrenergic system within the central nucleus of the amygdala in naloxone-precipitated morphine withdrawal-induced conditioned place aversion in rats. *Psychopharmacology*.

[50] Cameron OG (2006). Anxious-depressive comorbidity: effects on HPA axis and CNS noradrenergic functions. *Essential Psychopharmacology*.

[51] Almela P, Victoria Milanés M, Luisa Laorden M (2009). Tyrosine hydroxylase phosphorylation after naloxone-induced morphine withdrawal in the left ventricle. *Basic Research in Cardiology*.

[52] Zhaohui Z, Jingzhu Z, Guipeng D (2012). Role of neuropeptide Y in regulating hypothalamus-pituitary-gonad axis in the rats treated with electro-acupuncture. *Neuropeptides*.

[53] Zhu D, Ma Q, Li C, Wang L (2000). Effect of stimulation of Shenshu point on the aging process of genital system in aged female rats and the role of monoamine neurotransmitters. *Journal of Traditional Chinese Medicine*.

[54] Grimm JW, Lu L, Hayashi T, Hope BT, Su T-P, Shaham Y (2003). Time-dependent increases in brain-derived neurotrophic factor protein levels within the mesolimbic dopamine system after withdrawal from cocaine: implications for incubation of cocaine craving. *Journal of Neuroscience*.

[55] Bolaños CA, Nestler EJ (2004). Neurotrophic mechanisms in drug addiction. *NeuroMolecular Medicine*.

[56] Karpova NN, Pickenhagen A, Lindholm J (2011). Fear erasure in mice requires synergy between antidepressant drugs and extinction training. *Science*.

[57] Liu S, Zhou W, Liu H, Yang G, Zhao W (2005). Electroacupuncture attenuates morphine withdrawal signs and c-Fos expression in the central nucleus of the amygdala in freely moving rats. *Brain Research*.

[58] Lu P-K, Lu GP, Lu DP, Lu WI (2004). Managing acute withdrawal syndrome on patients with heroin and morphine addiction by acupuncture therapy. *Acupuncture and Electro-Therapeutics Research*.

[59] Chuang C-M, Hsieh C-L, Li T-C, Lin J-G (2007). Acupuncture stimulation at Baihui acupoint reduced cerebral infarct and increased dopamine levels in chronic cerebral hypoperfusion and ischemia-reperfusion injured Sprague-Dawley rats. *The American Journal of Chinese Medicine*.

[60] Yang CH, Lee BB, Jung HS, Shim I, Roh PU, Golden GT (2002). Effect of electroacupuncture on response to immobilization stress. *Pharmacology Biochemistry and Behavior*.

[61] Liu Q, Li B, Zhu H-Y, Wang Y-Q, Yu J, Wu G-C (2011). Glia atrophy in the hippocampus of chronic unpredictable stress-induced depression model rats is reversed by electroacupuncture treatment. *Journal of Affective Disorders*.

[62] Contet C, Filliol D, Matifas A, Kieffer BL (2008). Morphine-induced analgesic tolerance, locomotor sensitization and physical dependence do not require modification of mu opioid receptor, cdk5 and adenylate cyclase activity. *Neuropharmacology*.

